# Exploratory factor analysis yields grouping of brain injury biomarkers significantly associated with outcomes in neonatal and pediatric ECMO

**DOI:** 10.1038/s41598-024-61388-6

**Published:** 2024-05-11

**Authors:** Victoria Huang, Jennifer Roem, Derek K. Ng, Jamie McElrath Schwartz, Allen D. Everett, Nikhil Padmanabhan, Daniel Romero, Jessica Joe, Christopher Campbell, George B. Sigal, Jacob N. Wohlstadter, Melania M. Bembea

**Affiliations:** 1 Department of Anesthesiology and Critical Care Medicine, Johns Hopkins University School of Medicine, 1800 Orleans Street, Bloomberg Suite 6321, Baltimore, MD 21287 USA; 2 Department of Epidemiology, Johns Hopkins Bloomberg School of Public Health, Baltimore, MD USA; 3 Department of Pediatrics, Johns Hopkins University School of Medicine, Baltimore, MD USA; 4 Meso Scale Discovery, Rockville, MD USA

**Keywords:** Extracorporeal membrane oxygenation, Child, Brain injury, Biomarkers, Neurologic outcome, Biomarkers, Brain injuries, Paediatrics, Therapeutics

## Abstract

In this two-center prospective cohort study of children on ECMO, we assessed a panel of plasma brain injury biomarkers using exploratory factor analysis (EFA) to evaluate their interplay and association with outcomes. Biomarker concentrations were measured daily for the first 3 days of ECMO support in 95 participants. Unfavorable composite outcome was defined as in-hospital mortality or discharge Pediatric Cerebral Performance Category > 2 with decline ≥ 1 point from baseline. EFA grouped 11 biomarkers into three factors. Factor 1 comprised markers of cellular brain injury (NSE, BDNF, GFAP, S100β, MCP1, VILIP-1, neurogranin); Factor 2 comprised markers related to vascular processes (vWF, PDGFRβ, NPTX1); and Factor 3 comprised the BDNF/MMP-9 cellular pathway. Multivariable logistic models demonstrated that higher Factor 1 and 2 scores were associated with higher odds of unfavorable outcome (adjusted OR 2.88 [1.61, 5.66] and 1.89 [1.12, 3.43], respectively). Conversely, higher Factor 3 scores were associated with lower odds of unfavorable outcome (adjusted OR 0.54 [0.31, 0.88]), which is biologically plausible given the role of BDNF in neuroplasticity. Application of EFA on plasma brain injury biomarkers in children on ECMO yielded grouping of biomarkers into three factors that were significantly associated with unfavorable outcome, suggesting future potential as prognostic instruments.

## Introduction

Extracorporeal membrane oxygenation (ECMO) is a technique that provides support in neonatal and pediatric patients with severe refractory cardiopulmonary failure or cardiac arrest. It is a potentially lifesaving intervention, and technological advancements over the last decades have made utilization increasingly prevalent in critical care medicine. The Extracorporeal Life Support Organization (ELSO) registry most recently reported that during the five-year period between 2018 and 2022, there were a total of 12,196 pediatric and 7575 neonatal ECMO cases at 557 centers. In these populations, the indications for ECMO spanned 31–51% for respiratory support, 38–47% for cardiac support, and 11–22% for extracorporeal cardiopulmonary resuscitation (ECPR)^1^. The overall rate of survival to hospital discharge was 59–61%, which encompassed 69–70% for respiratory indications, 52–60% for cardiac indications, and 40–47% for ECPR indications^1^.

Acute neurologic injury is one of the major complications of ECMO, occurring in 13–36% of children on ECMO support^2–4^. Acute neurologic injury during ECMO includes intracranial hemorrhage, thromboembolic stroke, hypoxic-ischemic injury, and seizures, and is associated with increased mortality^2^ and long-term neurologic disability among survivors^5,6^. While morbidity and mortality on ECMO are certainly not limited to neurologic etiologies, neurologic injury is one of the main drivers of outcome in the neonatal and pediatric ECMO populations^3^.

The pathophysiology of neurologic injury in critically ill patients requiring ECMO support is complex and not yet well understood. Several neuromonitoring methods are used for early diagnosis of neurologic injury, therapy guidance, avoidance of secondary insults, and neuroprognostication^3,4^. However, it is likely that only multimodal monitoring can capture said complexity. Plasma brain injury biomarkers have been proposed as additions to multimodal monitoring and have been previously shown to be associated with neurologic injury in critically ill children, including those on ECMO support. These include biomarkers of primary structural damage but also of secondary cascade of injury and repair in the brain^7^, such as markers of neuronal injury: neuron-specific enolase (NSE)^8,9^, of neuroregeneration: brain-derived neurotrophic factor (BDNF)^9^, of astrocytic injury: glial fibrillary acidic protein (GFAP)^9,10^ and S100β^8,9,11^, and of neuroinflammation: monocyte chemoattractant protein 1 (MCP1)^9^.

Additionally, biomarkers that are relevant in neonatal or adult brain injury and thus may have pediatric implications include: platelet-derived growth factor receptor-beta (PDGFRβ)^12^, visinin-like protein 1 (VILIP-1)^13,14^, matrix metalloproteinase 9 (MMP-9)^15–19^, neurogranin (NRGN)^20,21^, creatine kinase B type (CK-BB)^11^, and von Willebrand factor (vWF)^22–24^. Lastly, biomarkers that have been identified in vitro or in animal models of brain injury but not yet established clinically, include neuronal pentraxin 1 (NPTX1)^25–28^ and peroxiredoxin-6 (PRDX6)^29,30^. Understanding the role of these biomarkers that represent multiple neuronal cell subtypes and neurologic injury pathways during neonatal and pediatric ECMO can potentially lead to improvements in neuromonitoring, identification of potential therapeutic targets, reduction in ECMO-related morbidity and mortality, and neuroprognostication among survivors.

In this study, we aimed to assess if use of exploratory factor analysis (EFA) on an expanded panel of brain injury biomarkers previously evaluated in various types of brain injury could lead to better understanding of the interplay of these biomarkers and their association with unfavorable outcomes and abnormal neuroimaging in critically ill children on ECMO support.

## Materials and methods

### Study population

This prospective observational cohort study enrolled neonatal and pediatric patients on ECMO support at two academic, quaternary care, urban, pediatric intensive care units between July 2010 and June 2015. Characteristics of the cohort have been previously described^5^. Informed consent was obtained from all parents within 24 h of ECMO initiation. A total of 99 children were enrolled in the parent study. Of these, 95 subjects had longitudinal blood samples obtained daily during the first 3 days of the ECMO course. Platelet-poor plasma aliquots that had not undergone any freeze–thaw cycles and had been stored at -80 °C in a freezer with continuous temperature monitoring were used for the current study. We collected data from neuroimaging reports of serial head ultrasounds obtained daily during the ECMO course in infants with open anterior fontanel as part of routine clinical protocols at the two participating institutions, and of brain computed tomography (CT) and/or brain magnetic resonance imaging (MRI) studies that were obtained based on clinical indications at the discretion of the clinical team, during the ECMO course and up to six weeks after decannulation. Clinical imaging reports noting abnormal findings were then classified as postasphyxial injury, arterial ischemic stroke, and intracranial hemorrhage, noting that imaging studies frequently detected more than one type of injury. Types of neuroimaging findings and time to first abnormal neuroimaging are presented in Supplemental Table 1. Pediatric Cerebral Performance Category (PCPC) scores at baseline and hospital discharge were ascertained from the medical records locally and in real-time by trained data abstractors who were blinded to biomarker results^31,32^. This study was approved by the Johns Hopkins Medicine and the Children’s National Institutional Review Boards at the two participating centers and performed in accordance with relevant guidelines and regulations.

### Measurement of brain injury biomarkers

Thirteen brain injury biomarkers were measured using chemiluminescent immunoassays (Meso Scale Discovery, Gaithersburg, MD). Of the 13 biomarkers, 11 were selected for the present study based on detectability in ≥ 50% of samples: brain-derived neurotrophic factor (BDNF), glial fibrillary acidic protein (GFAP), monocyte chemoattractant protein 1 (MCP1), matrix metalloproteinase 9 (MMP-9), neurogranin (NRGN), neuronal pentraxin 1 (NPTX1), neuron-specific enolase (NSE), platelet-derived growth factor beta (PDGFRβ), S100β, visinin-like protein 1 (VILIP-1), and von Willebrand factor (vWF). Markers not included in the analysis were peroxiredoxin-6 (PRDX6), which had more than half of samples above the upper limit of quantification, and creatine kinase B type (CKBB), for which the quantity of plasma available was insufficient to assay in all participants.

### Descriptive analysis of biomarkers

Of all available samples from a given participant, the highest value, or peak, of any given biomarker measured daily for the first 3 days of ECMO support was used for EFA. Biomarkers had right-skewed distributions and were therefore natural log-transformed. Seventy-two of 295 biomarker observations lay above their upper limit of quantification (ULOQ), all related to 5 (GFAP, MCP1, MMP-9, S100β, VILIP-1) of the 11 biomarkers included in the analysis. To utilize EFA, which requires numerical values for all data, we imputed values for these 72 observations using truncated log-normal distributions for each biomarker. Specifically, we fit a normal distribution to the observed data, extrapolated the curve above the ULOQ, and drew values randomly from the distribution above the ULOQ.

### EFA methods

We used EFA to detect combinations of biomarkers that indicated unique processes agnostic to covariates and outcomes of interest. EFA identifies groups of markers that are highly correlated and thus likely to reflect the same process. Log-transformed biomarker values (observed and imputed) were standardized to create distributions with a mean of 0 and a standard deviation of 1. Principal components analysis was used to produce proportion criteria and create a scree plot used to reduce the brain injury biomarkers to an optimal number of factors that retained the most amount of total variance in the original variables. The factors were derived using generalized weighted least squares methods and oblique rotation on the peak measurements to transform the factors and obtain factor loadings, which represent the strength and direction of the relationship between the biomarkers and the underlying (latent) relationships. For factor interpretation, only biomarkers with factor loadings ≥ 0.3 were considered; however, the final factor scores were based on all variables. The standardized factor scores for each participant were derived using the tenBerge regression scoring method, which preserves the correlation between factors for an oblique solution^33^.

The factors were then included as dependent variables in linear regression models to evaluate associations with clinical characteristics including participant age, sex, and ECMO indication. Next, the factors were included as independent variables in multivariable logistic regression models. The primary outcome for the first set of models was a composite unfavorable outcome, defined as in-hospital mortality or decline in neurofunctional status defined as discharge PCPC > 2 with decline ≥ 1 from baseline PCPC^5^. The secondary outcome for the second set of logistic models was new abnormal neuroimaging during the ECMO course or within 6 weeks of ECMO decannulation, evaluated among the subset of patients with available neuroimaging. These logistic regression models included each factor separately and summarized the association between each factor and unfavorable outcome, unadjusted and adjusted for age, sex, and ECMO indication. All analyses were performed using R 3.6.0 (R Core Team, Vienna, Austria).

## Results

Demographic and clinical characteristics of the parent cohort (n = 99) have been previously described^5^. Summary characteristics of the 95 participants included in this analysis are presented in Table 1.

**Table 1:** Demographic and clinical characteristics of children supported on ECMO^a^.

	Overall (n = 95)	Favorable (n = 43)	Unfavorable^b^ (n = 52)
Demographic characteristics			
Male	52 (55)	21 (49)	31 (6)
Age group			
Neonate, < 1 mo	44 (46)	24 (56)	20 (38)
Infant, 1 mo-< 1 y	24 (25)	7 (16)	17 (33)
Child, 1-< 12 y	19 (20)	7 (16)	12 (23)
Adolescent, ≥ 12 y	8 (8)	5 (12)	3 (6)
Weight, kg	4.0 [3.1, 11.9]	4.0 [3.2, 11.9]	4.0 [3.0, 11.7]
ECMO characteristics			
Primary indication for ECMO			
Respiratory	31 (33)	22 (51)	9 (17)
Non-respiratory	64 (67)	21 (49)	43 (83)
ECMO mode: venoarterial	88 (93)	38 (88)	50 (96)
ECMO duration, days	4.9 [3.2, 10.2]	4.6 [3.5, 7.5]	5.4 [3.2, 12.5]
Neuroimaging (n = 84)			
Neuroimaging during or within 6 weeks post-ECMO decannulation			
Daily HUS during or post-ECMO	34 (40)	11 (31)	23 (48)
Daily HUS during ECMO, brain CT during or post-ECMO, and/or brain MRI post-ECMO	32 (38)	18 (50)	14 (29)
At least one brain CT during or post-ECMO and/or brain MRI post-ECMO, without HUS	18 (21)	7 (19)	11 (23)
Outcomes			
Composite unfavorable outcome^b^	52 (55)	NA	52 (100)
In-hospital mortality	42 (44)	NA	42 (81)
Abnormal neuroimaging (n = 84)	42 (50)	15 (42)	27 (56)

ECMO, extracorporeal membrane oxygenation, PCPC, Pediatric Cerebral Performance Category, HUS, head ultrasound, CT, computed tomography, MRI, magnetic resonance imaging.

^a^Continuous variables are presented as medians [P25, P75] and categorical variables as counts (frequencies).

^b^Composite unfavorable outcome: in-hospital mortality or decline in neurofunctional status defined as discharge PCPC > 2 with decline ≥ 1 from baseline PCPC among survivors.

Supplemental Fig. 1 displays the distribution of daily plasma levels of each biomarker during the first 3 days of ECMO support. The distributions of peak plasma biomarker levels are summarized in Supplemental Table 2, which also includes counts and frequencies of biomarker levels above the ULOQ which occurred in 5 biomarkers (GFAP, MCP1, MMP-9, S100β, and VILIP-1). These counts above the ULOQ correspond to the 72 observations that were imputed using the log-normal models shown in Supplemental Fig. 2.

Correlations among the standardized log-transformed peak plasma biomarker levels are shown in Table 2, with moderately strong relationship (correlation coefficient ≥ 0.30) noted for: GFAP and S100β, MCP1, VILIP-1, NSE, BDNF, NRGN; S100β and MCP1, VILIP-1, NSE; MCP1 and VILIP-1, NSE; VILIP-1 and NSE; NSE and NRGN; BDNF and MMP-9; vWF and PDGFRβ, NPTX1; and PDGFRβ and NPTX1. We also observed a moderately strong inverse correlation between VILIP-1 and MMP-9. Factor loading and variance explained results from the EFA are shown in Table 3. Three brain injury factors were identified, accounting for 44% of the total variance in the biomarker data. Factor 1 was characterized by the biomarkers GFAP, S100β, MCP1, VILIP-1, NSE, BDNF, and NRGN; factor 2 by NPTX1, vWF, and PDGFRβ; and factor 3 by BDNF and MMP-9.

**Table 2:** Pearson correlations among the standardized log-transformed peak plasma biomarker levels (n = 95).

Marker	Pearson correlation										
	GFAP	S100β	MCP1	VILIP-1	NSE	BDNF	NRGN	vWF	PDGFRβ	NPTX1	MMP-9
GFAP	1.00	0.50 ^a,b^	0.41 ^a,b^	0.42 ^a,b^	0.47 ^a,b^	0.32 ^a,c^	0.30 ^a,c^	0.20^c^	0.15	0.16	− 0.08
S100β	–	1.00	0.47 ^a,b^	0.44 ^a,b^	0.33 ^a,c^	0.18	0.10	0.26^c^	0.20	0.17	− 0.14
MCP1	–	–	1.00	0.40 ^a,b^	0.33 ^a,c^	0.10	0.24^c^	0.12	0.14	0.17	− 0.23^c^
VILIP-1	–	–	–	1.00	0.41 ^a,b^	0.12	0.20^c^	0.18	0.12	0.27^c^	− 0.32 ^a,c^
NSE	–	–	–	–	1.00	0.24^c^	0.33 ^a,c^	0.29^c^	0.13	0.29^c^	− 0.26^c^
BDNF		–	–	–	–	1.00	0.24^c^	0.04	0.05	0.09	0.35 ^a,b^
NRGN	–	–	–	–	–	–	1.00	0.18	0.29^c^	0.23^c^	0.13
vWF	–	–	–	–	–	–	–	1.00	0.30 ^a,c^	0.46 ^a,b^	0.00
PDGFRβ	–	–	–	–	–	–	–	–	1.00	0.36 ^a,b^	− 0.05
NPTX1	–	–	–	–	–	–	–	–	–	1.00	− 0.02
MMP-9	–	–	–	–	–	–	–	–	–	–	1.00

BDNF, brain-derived neurotrophic factor; GFAP, glial fibrillary acidic protein; MCP1, monocyte chemoattractant protein 1; MMP-9, matrix metalloproteinase 9; NRGN, neurogranin, NPTX1, neuronal pentraxin 1; NSE, neuron-specific enolase; PDGFRβ, platelet derived growth factor receptor beta; VILIP-1, visinin-like protein 1, vWF, von Willebrand factor.

^a^Correlation coefficient ≥ 0.30 (moderately strong relationship).

^b^*p* < 0.001.

^c^0.001 ≤ *p* < 0.05.

**Table 3:** Factor loadings showing the strength of relationship between factors and individual biomarkers (n = 95).

Biomarker	Factor Loadings for Brain Injury Biomarkers		
	1	2	3
GFAP	**0.76**	− 0.02	0.08
S100β	**0.61**	0.05	− 0.09
MCP1	**0.58**	0.01	− 0.16
VILIP-1	**0.57**	0.09	− 0.24
NSE	**0.54**	0.18	− 0.08
BDNF	**0.45**	− 0.06	**0.53**
NRGN	**0.30**	0.27	0.24
vWF	0.02	**0.63**	0.01
PDGFRβ	0.01	**0.53**	0.02
NPTX1	− 0.01	**0.73**	0.01
MMP-9	− 0.14	0.03	**0.83**
Variance explained, %	21	13	10
Cumulative variance	21	34	44

BDNF, brain-derived neurotrophic factor; GFAP, glial fibrillary acidic protein; MCP1, monocyte chemoattractant protein 1; MMP-9, matrix metalloproteinase 9; NRGN, neurogranin, NPTX1, neuronal pentraxin 1; NSE, neuron-specific enolase; PDGFRβ, platelet-derived growth factor receptor beta; VILIP-1, visinin-like protein 1, vWF, von Willebrand factor.

Bold indicates salient factors using criterion of factor loadings ≥ 0.3.

To evaluate the relationship between brain injury biomarker factor scores and exposures (age, sex, and ECMO indication), we performed univariable linear regression analyses where each factor score was treated as the dependent variable while the exposures were the independent variables. Factor 1 score was associated with ECMO indication: participants with non-respiratory ECMO indications had mean factor 1 scores + 0.78 higher (*p* < 0.001) than participants with respiratory ECMO indications. Factor 2 and 3 scores were not significantly associated with any of the exposures (Table 4).

**Table 4:** Unadjusted associations of the three brain injury biomarker factors with the exposures of interest (n = 95).

Exposure	Brain injury biomarker factors^a^					
	1		2		3	
	Est (95% CI)	*p*	Est (95% CI)	*p*	Est (95% CI)	*p*
Sex: Male vs Female (ref)	0.31 (− 0.09, 0.72)	0.131	0.04 (− 0.37, 0.45)	0.849	− 0.08 (− 0.49, 0.33)	0.708
Age: Neonate vs Non-neonate (ref)	− 0.23 (− 0.63, 0.18)	0.276	0.29 (− 0.12, 0.69)	0.166	− 0.32 (− 0.73, 0.08)	0.119
ECMO indication: Non-respiratory vs Respiratory (ref)	0.78 (0.37, 1.18)	< 0.001	0.34 (− 0.09, 0.77)	0.118	− 0.19 (− 0.62, 0.25)	0.390

ECMO, extracorporeal membrane oxygenation; CI, confidence interval.

^a^Brain injury biomarker factors:

Factor 1: GFAP, S100β, MCP1, VILIP-1, NSE, BDNF, NRGN.

Factor 2: NPTX1, vWF, PDGFRβ.

Factor 3: BDNF, MMP-9.

**Figure 1:**
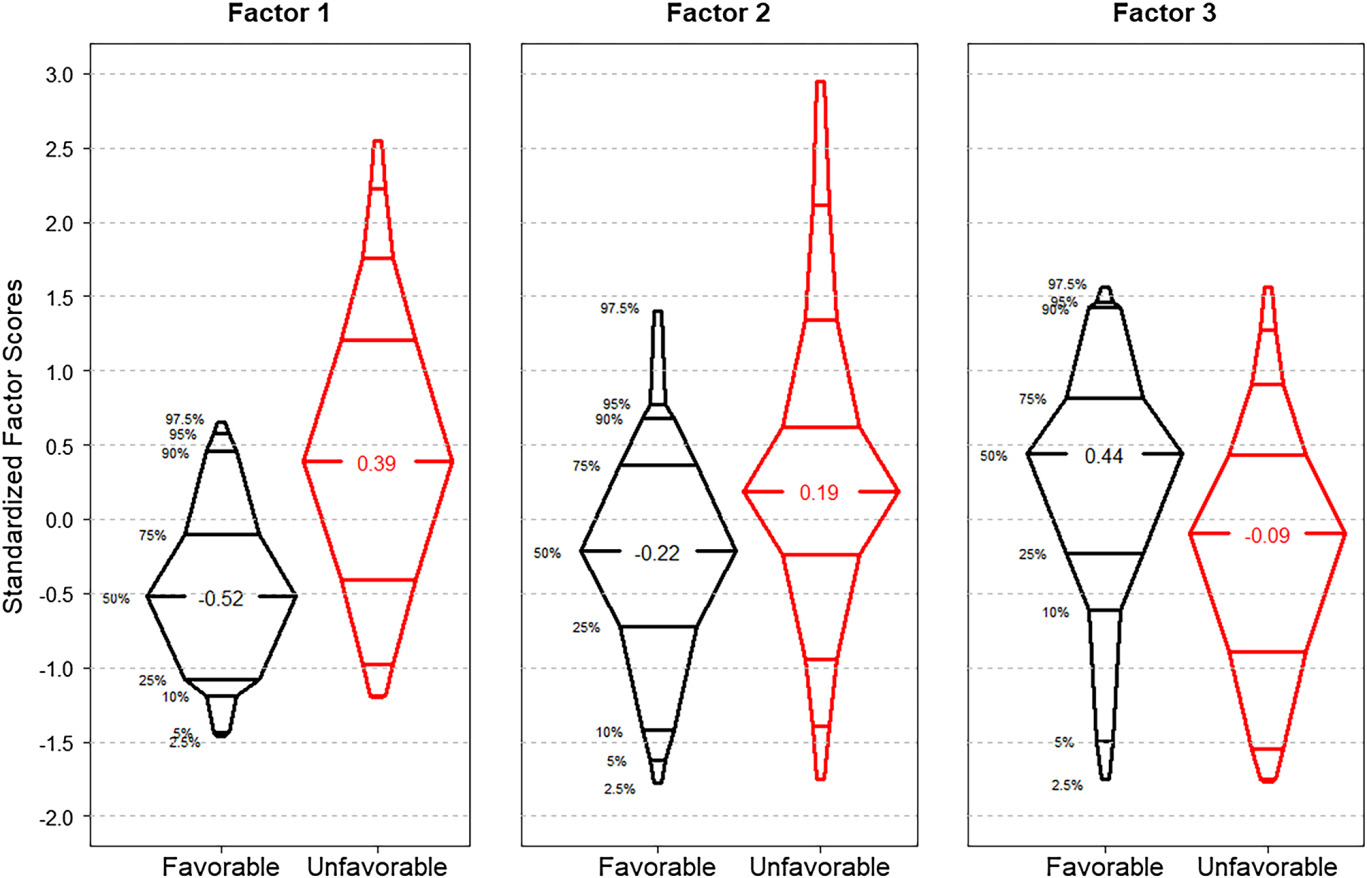
Distributions of the three brain injury biomarker factors by outcome (n = 95). The black plots show those with favorable outcome (n = 43), and the red plots show those with unfavorable outcome (n = 52). Wilcoxon rank sum comparisons between factor scores for favorable versus unfavorable outcomes rendered: *p* < 0.001 for Factor 1, *p* = 0.033 for Factor 2, and *p* = 0.008 for Factor 3.

The distributions of all three brain injury biomarker factor scores by unfavorable versus favorable outcome are graphically displayed in Fig. 1. These distributions indicate that an unfavorable outcome had higher median factor 1 and 2 scores compared to a favorable outcome (+ 0.39 versus -0.52, *p* < 0.001, and + 0.19 versus -0.22, *p* = 0.033, respectively). Conversely, lower median factor 3 scores were seen in unfavorable outcomes compared to favorable outcomes (-0.09 versus + 0.44, *p* = 0.008).

Table 5 presents results from multivariable logistic regression analyses where the composite unfavorable outcome was the dependent variable, and each brain injury biomarker factor score was the exposure of interest in separate models. Brain injury factors 1 and 2 were associated with higher odds of unfavorable outcome (adjusted OR 2.88, 95% CI 1.61–5.66, and adjusted OR 1.89, 95% CI 1.12–3.43, respectively), while brain injury factor 3 was associated with lower odds of unfavorable outcome (adjusted OR 0.54, 95% CI 0.31–0.88). The results of the full multivariable logistic regression models are presented in Supplemental Table 3.

**Table 5:** Multivariable model for association of the three brain injury biomarker factors with unfavorable outcome at hospital discharge^a^ (n = 95).

Brain injury biomarker factors^c^	Unadjusted		Adjusted^b^	
	OR (95% CI)	*p*	OR (95% CI)	*p*
1	3.33 (1.94, 6.32)	< 0.001	2.88 (1.61, 5.66)	0.001
2	1.75 (1.12, 2.89)	0.020	1.89 (1.12, 3.43)	0.025
3	0.59 (0.36, 0.91)	0.021	0.54 (0.31, 0.88)	0.020

^a^Unfavorable outcome at hospital discharge is defined as in-hospital mortality or discharge Pediatric Cerebral Performance Category (PCPC) > 2 with decline ≥ 1 point from baseline PCPC.

^b^Adjusted for age, sex, and ECMO indication.

^c^Brain injury biomarker factors:

Factor 1: GFAP, S100β, MCP1, VILIP-1, NSE, BDNF, NRGN.

Factor 2: NPTX1, vWF, PDGFRβ.

Factor 3: BDNF, MMP-9.

Among the subset of 84 participants who had neuroimaging studies completed during ECMO or within 6 weeks after ECMO decannulation, factor 1 was associated with higher odds of abnormal neuroimaging (adjusted OR 2.38, 95% CI 1.38–4.45). Factors 2 and 3 were not significantly associated with abnormal neuroimaging. The results of these multivariable logistic regression models are presented in Supplemental Table 4. Of note, abnormal neuroimaging was not statistically associated with the composite primary outcome, unadjusted (OR 1.80, 95% CI 0.76–4.37) or when adjusting for age, sex, and ECMO indication (adjusted OR 1.45, 95% CI 0.56–3.80).

## Discussion

In this two-center prospective observational study of 95 neonatal and pediatric patients on ECMO, we found that 11 circulating biomarkers could be grouped via EFA as a data reduction technique. Specifically, biomarker levels clustered together in three distinct brain injury factors that are biologically plausible based on what is currently known about originating cells and function of each of the biomarkers studied. More importantly, the results indicate that these factors were associated with unfavorable outcome at hospital discharge and new abnormal neuroimaging during or within 6 weeks after ECMO decannulation.

Brain injury factor 1 grouped the following biomarkers: GFAP, S100β, MCP1, NSE, BDNF, VILIP-1, and NRGN. This group encompasses biomarkers of cellular brain injury: neuronal injury, astrocytic injury, and neuroinflammation. To first address markers of neuronal injury, NSE is a glycolytic enzyme localized mostly in neuronal cytoplasm. It has been previously associated with unfavorable outcomes after pediatric cardiac arrest^34^. BDNF is a member of the neurotrophins family of growth factors and mediates neuronal growth, differentiation, regeneration, and survival. It has been implicated in providing neuroplasticity and neuroprotection through reduction in secondary brain injury. BDNF has been associated with pediatric traumatic brain injury (TBI)^35^, favorable outcomes in neonatal hypoxic-ischemic encephalopathy (HIE)^36,37^, and decreased functional impairment in pediatric neurocritical illness^38^. Next, to address markers of astrocytic injury, GFAP is a cytoskeletal filament protein found in mature astrocytes, and its expression is increased during reactive astrogliosis after neurologic injury^39^. It has been shown to predict neurologic injury and outcomes in pediatric patients on cardiopulmonary bypass^40,41^, in pediatric patients with sickle cell disease^42^ and severe TBI^43^, and in HIE^37,44,45^. S100β is a calcium-binding protein localized predominately in the cytoplasm of astrocytes and involved in neuronal growth and survival^12,48^ and has been associated with unfavorable outcome after pediatric cardiac arrest^34^ and in neonatal HIE^46^. Finally, to address neuroinflammation, MCP1 is a small cytokine in the CC chemokine family that plays a key role in recruiting immune factors and cells to sites of inflammation. In the central nervous system, it is found in neurons, astrocytes, microglia, and infiltrating macrophages and thus involved in acute neuroinflammation, neuronal injury and death. MCP1 has been shown to be elevated after neonatal HIE^47^. These five biomarkers (GFAP, S100β, MCP1, NSE, BDNF) have all been previously studied in pediatric ECMO. GFAP, S100β, NSE and MCP1 have been associated with neurologic injury and unfavorable outcomes in pediatric ECMO^9,48^. NSE has also been associated with abnormal neuroimaging in these studies^9^. BDNF’s neuroprotective factors have also been previously investigated in pediatric ECMO, but no studies have found associations with survival, outcomes, or neuroimaging^9^*.* Together, these five biomarkers represent three major categories of neurologic injury pathophysiology—neuronal injury, astrocytic injury, and neuroinflammation—and thus it is fitting for them to be agnostically grouped into one factor.

Two less studied biomarkers, VILIP-1 and NRGN, were also grouped into brain injury factor 1. VILIP-1 is a neuronal calcium sensor protein that has been studied in adult neurodegenerative diseases^49^, stroke^14^, and TBI^13^. The only clinical study in pediatrics found that serum and cerebrospinal fluid levels of VILIP-1 were associated with acute encephalopathy with biphasic seizures and late reduced diffusion^50^. NRGN is a brain-specific postsynaptic calmodulin-binding protein involved in the protein kinase C signaling pathway. Elevated levels have been observed in children with sickle cell disease and stroke^51^ and associated with worse neonatal HIE grades and developmental outcomes^37^. The addition of these two biomarkers with more well-established markers of brain injury in one grouped factor suggests that they may warrant further investigation in pediatric brain injury.

The second brain injury factor grouped: vWF, PDGFRβ, and NPTX1. Interestingly, two of the three markers are involved in vascular processes (vWF and PDGFRβ). vWF is a glycoprotein integrally involved in hemostasis through platelet and collagen adhesion and the intrinsic coagulation cascade. It has been previously studied as a marker of endothelial activation and injury in adults with TBI with mixed results^22–24^. PDGFRβ is a growth factor receptor that is released with pericyte damage and thus has been used as an indicator of microvascular injury and blood–brain barrier disruption in adult TBI, stroke, spinal cord injury, multiple sclerosis, and glioblastoma^12^. NPTX1 is a neuronal pentraxin protein preferentially secreted at excitatory synapses and has a role in regulating mitochondria-driven neuronal death in hypoxic-ischemic animal models^25–28^. None of these three markers have been previously studied in pediatric neurologic injury or critical illness. However, given the association with unfavorable outcomes in this study, they may warrant further investigation into vascular-related pathophysiology in pediatric brain injury.

Brain injury factor 3 grouped MMP-9 and BDNF. This is fitting as BDNF has been shown to upregulate MMP-9, a proteinase involved in extracellular matrix degradation, in terms of expression and enzymatic activity^52^. In turn, MMP-9 helps convert pro-BDNF to its activated form. MMP-9 has been previously associated in adult ischemic stroke severity, lesion volume, and hemorrhagic conversion^15–18^. It has not been as well studied in pediatrics, though elevated levels have been found in neonatal encephalopathy^19^ and in neuroinflammatory conditions^53^. This factor’s association with lower levels of unfavorable neurologic outcomes in neonatal and pediatric ECMO is supported by the previously discussed neuroprotective properties of BDNF. Interestingly, this suggested neuroprotective property of MMP-9 stands in contrast to previously demonstrated pathogenic properties of other proteases, such as calpain, in many neuropathologies including neonatal encephalopathy^54–56^. Additionally, the agnostic grouping of these two biomarkers involved in the same signaling pathway supports that EFA did in fact group combinations that indicate a unique pathophysiologic process.

In summary, EFA agnostically grouped 11 biomarkers into three factors that have compelling shared properties and appear to cluster together, with factor 1 represented by markers of cellular injury, factor 2 represented by markers of microvascular injury, and factor 3 represented by the BDNF and MMP-9 cellular pathway. This study provides insight into a diverse set of biomarkers that have had varying degrees of prior investigation in neonatal and pediatric ECMO and critical illness. For GFAP, S100β, and NSE, we support previously published associations with outcomes in pediatric ECMO. While BDNF and its neuroprotective role have been previously demonstrated, this is the first study to support the role in neonatal and pediatric ECMO. Additionally, we newly describe associations of VILIP-1, NRGN, vWF, PDGFRβ, and MMP-9, which have only been previously studied in infant, pediatric, or adult brain injury and not in neonatal and pediatric ECMO. We also newly describe clinical associations of NPTX1, which has only been previously studied in in vitro and animal models of brain injury. The agnostic grouping suggests that further investigations into clinical or biochemical associations of biomarkers within each factor may be of interest.

This study had several limitations. First, in our study population prior to ECMO, 20% had a pre-existing neurologic diagnosis, 40% had cardiac arrest, and 21% of children with available neuroimaging had acute abnormal findings^5^. Given this high prevalence of pre-ECMO neurologic conditions, it is difficult to specifically attribute these biomarker patterns to neurologic injury in critical illness versus neurologic injury on ECMO. Additionally, in this study, neuroimaging was obtained based on clinical indications and protocols, with head ultrasounds obtained daily for infants and brain CTs obtained when the clinical team had concerns for an acute neurological event. Given that neuroimaging cannot be consistently captured in a standardized manner in this patient population, the exact timing of an acute neurological event cannot be ascertained, with imaging lagging the actual time of onset of the event. Also, for this reason, abnormal neuroimaging is generally deemed not informative as a primary outcome; it was thus treated as a secondary outcome in our study. Furthermore, in the neonatal and pediatric ECMO population, it has been shown that abnormal neuroimaging findings during ECMO have limited associations with long-term neurodevelopmental outcomes in survivors^3^. Lastly, the definition of unfavorable outcome based on PCPC lacks granularity, however ongoing and future studies of blood-based biomarkers will continue to evaluate long-term outcomes as they relate to specific patterns of injury during critical illness with ECMO support.

## Conclusions

In this two-center prospective observational study of neonatal and pediatric ECMO, we found that 11 circulating biomarkers could be grouped via EFA to suggest three brain injury factors. The first brain injury factor grouped markers of cellular brain injury. Higher levels were associated with unfavorable survival and neurofunctional outcomes as well as with new abnormal neuroimaging. The second brain injury factor grouped markers related to vascular processes. Higher levels were also associated with unfavorable outcome. Lastly, the third brain injury factor grouped the BDNF and MMP-9 cellular pathway. This pathway was associated with neuroprotective properties, with lower levels associated with unfavorable outcome.

### Supplementary Information

Supplementary Information.

## Data Availability

The data used in this study are not publicly available because the study sites retain data ownership, and the data contain information that can compromise the privacy of research participants. MMB can be contacted to request data from this study.
