# Supplementary material for: Exploratory factor analysis yields grouping of brain injury biomarkers significantly associated with outcomes in neonatal and pediatric ECMO
Source: Sci Rep. 2024 May 11;14:10790. doi: 10.1038/s41598-024-61388-6 (PMC11088671; doi:10.1038/s41598-024-61388-6)
Supplement: Supplementary file 1 — Supplementary Information. [file 41598_2024_61388_MOESM1_ESM.docx]

**Exploratory Factor Analysis Yields Grouping of Brain Injury Biomarkers Significantly**

**Associated with Outcomes in Pediatric ECMO**

Victoria Huang, MD; Jennifer Roem, MS; Derek K Ng, PhD; Jamie McElrath Schwartz, MD;

Allen D Everett, MD; Nikhil Padmanabhan, MS; Daniel Romero, BS; Jessica Joe, BS; Christopher Campbell, MD, PhD; George B Sigal, PhD; Jacob N Wohlstadter, BS; Melania M Bembea, MD, PhD

**Supplemental Digital Content**

**Table of Contents**

| **Content** | **Pages** |
| --- | --- |
| **Supplemental Table 1.** Distributions of neuroimaging findings and time of first abnormal neuroimaging. | 2 |
| **Supplemental Figure 1.** Distributions of biomarkers for the first three days after ECMO initiation. | 3 |
| **Supplemental Table 2.** Distributions of peak plasma biomarker levels, with the upper limit of quantification shown. | 4 |
| **Supplemental Figure 2.** Cumulative incidence functions displaying distribution of peak plasma biomarker levels. | 5 |
| **Supplemental Table 3.** Multivariable models for association of the three brain injury biomarker factors with unfavorable outcome at hospital discharge. | 6 |
| **Supplemental Table 4.** Multivariable models for association of the three brain injury biomarker factors with abnormal neuroimaging. | 7 |

**Supplemental Table 1.** Distributions of neuroimaging findings (n=84) and time of first abnormal neuroimaging. Neuroimaging completed during or within 6 weeks post-ECMO decannulation.

|  | Neuroimaging done (n=84)^a^ | Time of first abnormal neuroimaging from ECMO start, hours^b^ |
| --- | --- | --- |
| No abnormalities | 42 (50%) | NA |
| Any abnormality | 42 (50%) | 138 [16, 393] |
| Arterial ischemic stroke | 18 (43%) | 155 [54, 397] |
| Intracranial hemorrhage | 30 (71%) | 112 [22, 363] |
| Postasphyxial injury | 16 (38%) | 16 [11, 49] |
| Types of neurologic injury | - | - |
| Arterial ischemic stroke only | 7 (8%) |  |
| Intracranial hemorrhage only | 10 (12%) |  |
| Postasphyxial injury only | 4 (5%) |  |
| Arterial ischemic stroke + Postasphyxial injury | 1 (1%) |  |
| Arterial ischemic stroke + Intracranial hemorrhage | 9 (11%) |  |
| Intracranial hemorrhage + Postasphyxial injury | 10 (12%) |  |
| Arterial ischemic stroke + Intracranial hemorrhage + Postasphyxial injury | 1 (1%) |  |

^a^ Presented as counts (frequencies)

^b^ Presented as medians [P25, P75]

**Supplemental Figure 1.** Distributions of biomarkers for the first three days after ECMO initiation (hours 0 to 27, n=92; hours 27 to 48, n=84; hours 48 to 72, n=73). c denotes the number of samples above the upper limit of detection. Black lines represent the median value for each day.


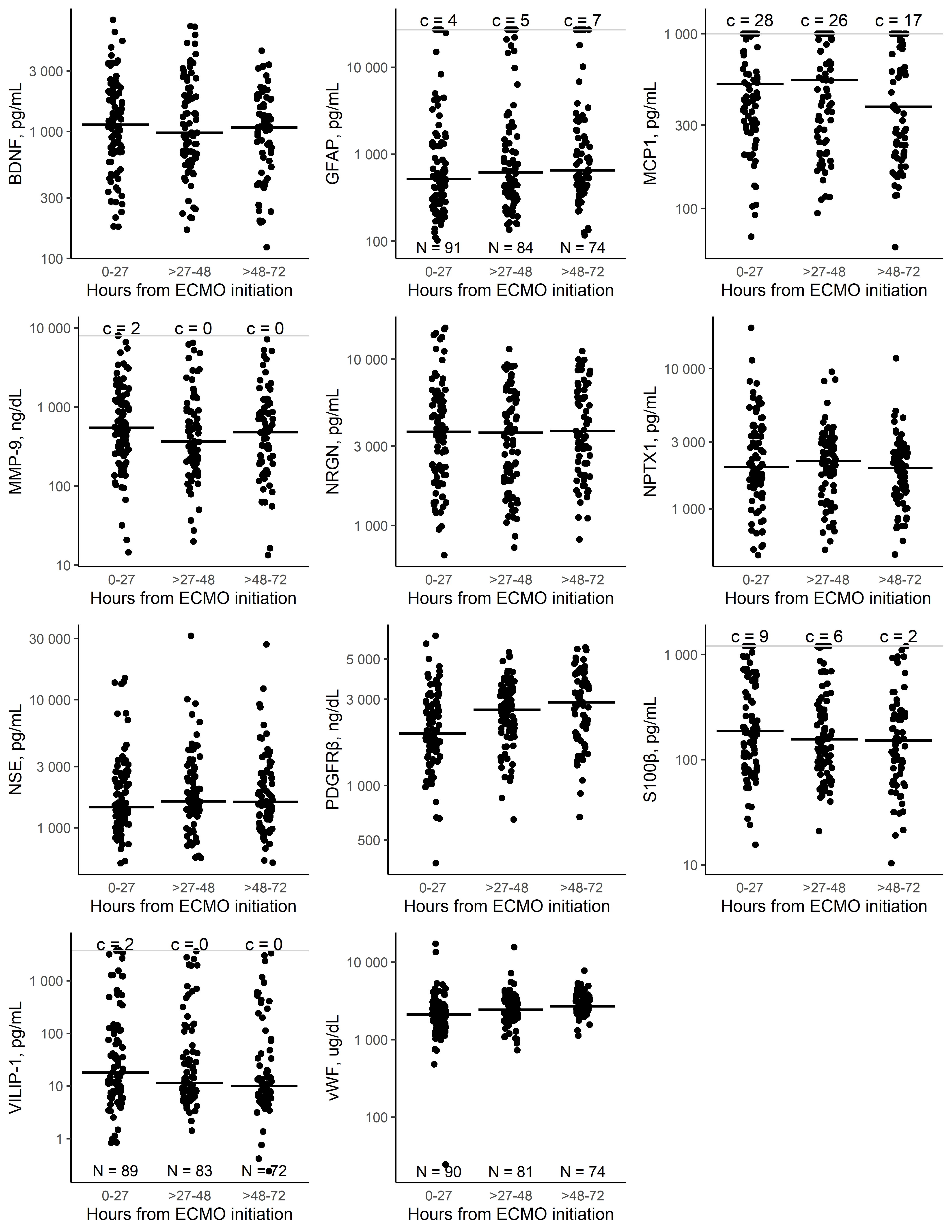


**Supplemental Table 2.** Distributions of peak plasma biomarker levels, with the upper limit of quantification shown (n=95).

| Marker | Median [IQR] | ULOQ | Proportion ≥ ULOQ |
| --- | --- | --- | --- |
| BDNF, pg/mL | 2009 [1355, 3067] | 65000 | 0% (0) |
| GFAP, pg/mL | 1239 [490, 3526] | 27000 | 16% (15) |
| MCP1, pg/mL | 764 [394, 1000] | 1000 | 42% (40) |
| MMP-9, ng/dL | 1087 [479, 2223] | 8000 | 2% (2) |
| NRGN, pg/mL | 5575 [3374, 7584] | 200000 | 0% (0) |
| NPTX1, pg/mL | 2512 [1667, 3827] | 45000 | 0% (0) |
| NSE, pg/mL | 2178 [1491, 3504] | 400000 | 0% (0) |
| PDGFRβ, ng/mL | 3111 [2196, 3907] | 50000 | 0% (0) |
| S100β, pg/mL | 248 [135, 765] | 1200 | 14% (13) |
| VILIP-1, pg/mL | 21 [9, 256] | 3750 | 2% (2) |
| vWF, μg/dL | 3159 [2440, 3961] | 40000 | 0% (0) |

Abbreviations: ULOQ, upper limit of quantification; BDNF, brain-derived neurotrophic factor; GFAP, glial fibrillary acidic protein; MCP1, monocyte chemoattractant protein 1; MMP-9, matrix metalloproteinase 9; NRGN, neurogranin, NPTX1, neuronal pentraxin 1; NSE, neuron-specific enolase; PDGFRβ, platelet derived growth factor receptor beta; VILIP-1, visinin-like protein 1, vWF, von Willebrand factor.

**
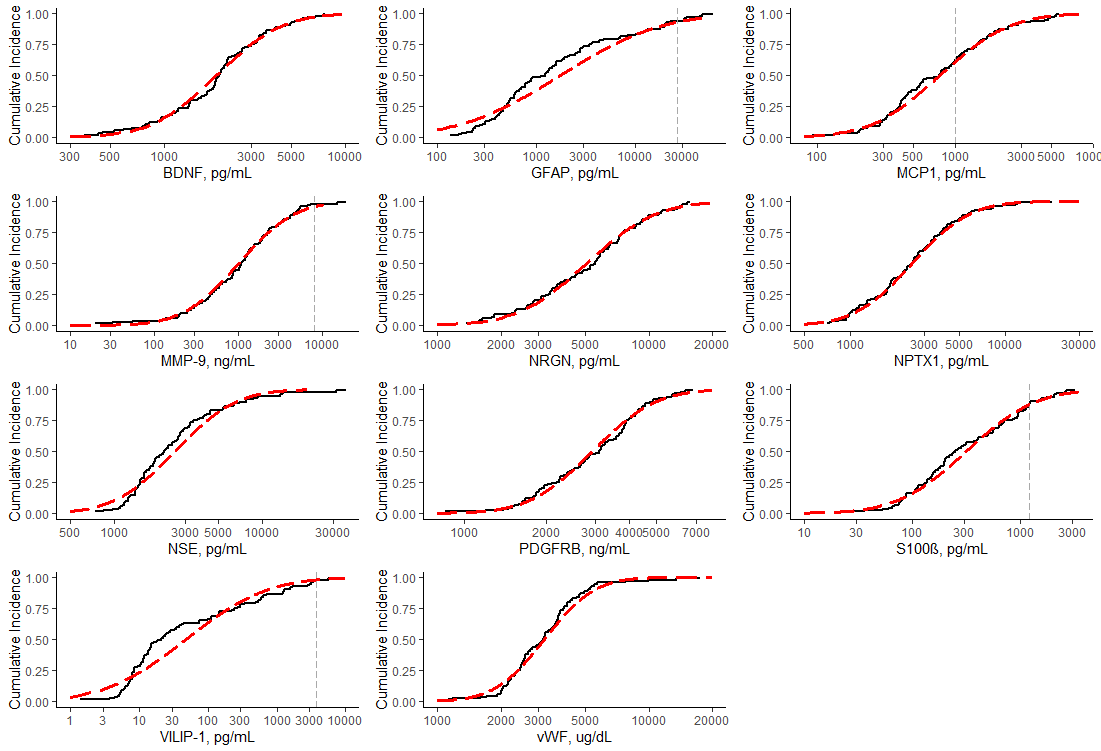
Supplemental Figure 2.** Cumulative incidence functions displaying distribution of peak plasma biomarker levels. Log-normal fit is shown by red dashed lines. For the 5 biomarkers where imputation was done (GFAP, MCP1, MMP-9, S100β, VILIP-1), the upper limit of quantification is shown by the vertical gray dashed line.

**Supplemental Table 3.** Multivariable models for association of the three brain injury biomarker factors with unfavorable outcome at hospital discharge^a^ (n=95)

|  | **Adjusted^b^ with Factor 1** | | **Adjusted^b^ with Factor 2** | | **Adjusted^b^ with Factor 3** | |
| --- | --- | --- | --- | --- | --- | --- |
|  | **OR (95% CI)** | **p** | **OR (95% CI)** | **p** | **OR (95% CI)** | **p** |
| **Brain injury biomarker factor^c^** | 2.88 (1.61, 5.66) | 0.001 | 1.89 (1.12, 3.43) | 0.001 | 0.54 (0.31, 0.88) | 0.020 |
| **Male sex** | 1.44 (0.53, 4.02) | 0.473 | 2.07 (0.79, 5.73) | 0.146 | 1.81 (0.70, 4.85) | 0.225 |
| **Neonate** | 0.58 (0.20, 1.64) | 0.308 | 0.42 (0.14, 1.19) | 0.107 | 0.45 (0.16, 1.24) | 0.126 |
| **Non-respiratory ECMO indication** | 2.57 (0.90, 7.59) | 0.081 | 3.48 (1.30, 9.82) | 0.015 | 3.91 (1.45, 11.15) | 0.008 |
| ^a^ Unfavorable outcome at hospital discharge is defined as in-hospital mortality or discharge Pediatric Cerebral Performance Category (PCPC) >2 with decline ≥1 point from baseline PCPC  ^b^ Adjusted for age, sex, and ECMO indication  ^c^ Brain injury biomarker factors:  Factor 1: GFAP, S100β, MCP1, VILIP-1, NSE, BDNF, NRGN  Factor 2: NPTX1, vWF, PDGFRβ  Factor 3: BDNF, MMP-9 | | | | | | |

**Supplemental Table 4.** Multivariable models for association of the three brain injury biomarker factors with abnormal neuroimaging^a^ (n=84)

|  | **Adjusted^b^ with Factor 1** | | **Adjusted^b^ with Factor 2** | | **Adjusted^b^ with Factor 3** | |
| --- | --- | --- | --- | --- | --- | --- |
|  | **OR (95% CI)** | **p** | **OR (95% CI)** | **p** | **OR (95% CI)** | **p** |
| **Brain injury biomarker factor^c^** | 2.38 (1.38, 4.45) | 0.003 | 1.24 (0.77, 2.07) | 0.385 | 1.34 (0.82, 2.25) | 0.256 |
| **Male sex** | 0.83 (0.30, 2.33) | 0.729 | 1.23 (0.48, 3.20) | 0.668 | 1.24 (0.49, 3.25) | 0.651 |
| **Neonate** | 0.61 (0.20, 1.81) | 0.373 | 0.44 (0.15, 1.23) | 0.121 | 0.52 (0.18, 1.46) | 0.216 |
| **Non-respiratory ECMO indication** | 0.88 (0.28, 2.73) | 0.823 | 1.21 (0.41, 3.57) | 0.723 | 1.28 (0.44, 3.76) | 0.653 |
| ^a^ Arterial ischemic stroke, intracranial hemorrhage, and/or postasphyxial brain injury during or within 6 weeks post-ECMO decannulation  ^b^ Adjusted for age, sex, and ECMO indication  ^c^ Brain injury biomarker factors:  Factor 1: GFAP, S100β, MCP1, VILIP-1, NSE, BDNF, NRGN  Factor 2: NPTX1, vWF, PDGFRβ  Factor 3: BDNF, MMP-9 | | | | | | |
